# Targeted microwave ablation of localised prostate cancer: Initial results of VIOLETTE trial

**DOI:** 10.1002/bco2.444

**Published:** 2024-10-30

**Authors:** Nicolas Barry Delongchamps, Alexandre Peltier, Eric Potiron, Franck Bladou, Julien Anract, Romain Diamand, Grégoire Robert, Aurel Messas, Roland Van Velthoven

**Affiliations:** ^1^ Department of Urology, Cochin Hospital, Assistance Publique – Hôpitaux de Paris Université Paris Cité Paris France; ^2^ Department of Urology, Jules Bordet Institute, Hôpital Universitaire de Bruxelles Université Libre de Bruxelles Brussels Belgium; ^3^ Department of Urology Clinique Urologique Nantes Atlantis Saint Herblain France; ^4^ Department of Urology, CHU de Bordeaux Université de Bordeaux Bordeaux France; ^5^ Department of Urology American Hospital Paris France

**Keywords:** cancer, focal therapy, microwave, organ‐based tracking, prostate

## Abstract

**Objective:**

The aim of this study was to assess the precision and safety of targeted microwave ablation (TMA) using organ‐based tracking (OBT) fusion, in patients with intermediate risk prostate cancer.

**Patients and method:**

We conducted a prospective, multicentric trial. Eligible patients had a prostate‐specific antigen (PSA) < 20 ng/mL, a magnetic resonance imaging (MRI)‐visible index tumour of Gleason score 3 + 4, with largest axis ≤15 mm and distant of at least 5 mm from the rectum and apex. TMA was performed with microwave needle applicator using OBT fusion, with a transperineal or a transrectal approach. In this interim analysis, we evaluated precision, safety, urinary and sexual outcomes, and PSA density kinetics.

**Results:**

At this point, 37 patients were treated in five centres. Median (interquartile range) age is 68 (63–72) years. Baseline median prostate volume and PSA are of 45 (34–57) mL and 8 (6.2–10.8) ng/mL, respectively. Median largest tumour axis on T2W MRI is of 11 mm (10–13). Patients were treated under general anaesthesia or conscious IV sedation in an outpatient setting. Anaesthesia had a median duration of 78 (66–90) min. A median number of 3 (2–4) 12‐W ablations of 2 to 5 min were performed per patient. After a median follow‐up of 6 (2.4–10) months, we observed 58 adverse events (AE) in 22 patients. These were of Common Terminology Criteria for Adverse Events (CTCAE) grade 1, 2 and 3 in 43 (74%), 13 (22%) and 2 (4%) cases. Six (15%) patients had an acute urinary retention, five of which considered as severe AE because of rehospitalisation. We did not observe any significant difference in International Prostate Symptom Score (IPSS), Male Sexual Health Questionnaire‐ejaculatory dysfunction (MSHQ‐EjD) and International Index of Erectile Function (IIEF5) from baseline to last follow‐up. Median PSA density evolved from 0.2 (0.1–0.3) at baseline to 0.1 (0.07–0.16) at 12 months.

**Conclusions:**

These preliminary results suggest that TMA using OBT fusion is precise and safe in patients with intermediate risk localised prostate cancer. Further inclusions and follow‐up are needed to assess oncological outcome.

## 1 INTRODUCTION

Focal therapy (FT) of localised prostate cancer (PCa) aims to eradicate clinically significant disease, thereby decreasing the risk of cancer progression, while preserving key structures that are essential for genitourinary function. This strategy has been driven by the development of multiparametric magnetic resonance imaging (mpMRI)^1^ and organ‐based tracking (OBT) MRI‐ultrasound fusion,^2^, ^3^ which now allows clinicians to detect and target precisely the main region of interest (ROI) generally defined as the index tumour.^4^, ^5^ A number of sources of energy have been employed so far to ablate the targeted area, but according to actualised guidelines, this treatment strategy remains experimental because of lack of robust oncological data.^6^, ^7^, ^8^

Targeted microwave ablation (TMA) is a novel FT modality involving targeted delivery of microwave energy under mpMRI/ultrasound guidance using 3D ultrasound and OBT fusion technology (Koelis Trinity). This new FT stands out from others by the fact that it is a global strategy, including not only a specific ablation modality but also and more importantly a comprehensive FT guidance tool: the OBT fusion system of the Trinity station allows to recall the location of the targeted biopsy as well as to visualise the MRI‐visible tumour. An additional software implanted in the station allows adapting in a real‐time manner the FT volume and shape that the surgeon aims to cover, including the margins. Four preliminary studies suggested the feasibility and safety of the technique, using the transrectal^9^, ^10^ or transperineal^11^, ^12^ approach. In the first‐in‐man pilot study, the precision of ablation was demonstrated on post‐operative mpMRI in eight out of 10 patients.^9^ The shape of the prostatic necrosis obtained was sharp, and its volume corresponded to what had been forecasted on pre‐clinical predictive ablation chart.

Prospective data on TMA are however still sparse, with no more than 40 patients evaluated and reported in the literature, and a follow‐up period of only 6 months. Oncological data are also needed to confirm the potential value of this FT to eradicate significant cancer. We thus undertook a European prospective phase II multicentric trial in France and in Belgium to assess the oncological value of TMA, as a primary objective, in patients with intermediate risk localised prostate cancer.

The present report is an interim analysis aiming to describe the precision of the technique and the early safety results from this multicentric evaluation.

## 2 PATIENTS AND METHOD

### 2.1 Study characteristics and endpoints

This prospective phase II interventional trial was approved by the French committee for medical and health ethics, and registered on ClinicalTrial.gov (NCT04582656).

Primary objective is to assess the treatment efficacy, with a primary outcome being to achieve a success rate of more than 80%. Treatment success is defined as no International Society of Urological Pathology (ISUP) ≥2 cancer in the treated area, measured with targeted and systematic biopsies performed 12 months after TMA.

Secondary objectives and endpoints are (1) intra‐ and post‐operative outcomes, including safety, assessed with prostatic mpMRI 7 days after procedure, adverse events (AEs) reporting and pain score evaluation; (2) urinary and sexual outcomes; (3) prostate‐specific antigen (PSA) kinetics; and (4) treatment zone shape on MRI at day 7, 6 and 12 months.

### 2.2 Patients inclusion

The inclusion criteria are the following: (1) age between 45 and 80 years; (2) patients with an intermediate‐risk PCa defined a PSA < 20 ng/mL and a unique clinically significant cancer focus of Gleason score 3 + 4 or ISUP 2, identified on a multiparametric MRI with a largest axis ≤15 mm, and distant of at least 5 mm from the rectum and the far apex, respectively.

### 2.3 Intervention

All procedures are performed under general anaesthesia and conscious IV sedation. The technique was described previously.^9^, ^10^, ^11^, ^12^ Urethral catheterisation is performed on a case‐by‐case basis to locate the urethra during the procedure. The ultrasound probe is inserted transrectally and held with a mechanical arm (Steady Pro™, Koelis, France). Ultrasound‐MRI image fusion is performed with OBT‐registration using Trinity™ platform (Koelis, France).^2^, ^13^ Microwave ablation is provided by the TATO™ generator (Biomedical, Firenze, Italy), through a single 17G needle inserted into the index lesion via a transperineal approach, or through a 18G needle via a transrectal approach, according to the investigator's decision. Duration and power of microwave application are set according to predictive ablation charts. For each setting, the Trinity platform displays the predictive ablated volume, allowing the investigator to choose the optimal position of the needle and decide on the number of ablations, in order to cover the entire index lesion with a treatment margin of at least 3 mm in all planes or directions.

### 2.4 Follow‐up

All patients are reviewed at day 7 and at 1, 3, 6, 9 and 12 months after surgery. Total PSA is collected at 3 and 6 months. International Prostate Symptom Score (IPSS), International Index of Erectile Function (IIEF5) and MSHQ‐EjD‐SF self‐administered questionnaires are collected at each visit. A multiparametric prostate MRI is performed at day 7, and at 6 and 12 months, and prostate volume is collected at these time points. The extent of necrosis at day 7 is evaluated with T1‐contrast enhanced weighted sequences. Prostate re‐biopsy is performed at 6 months only in case of untreated or relapsing tumour suspicion on mpMRI. AEs are reported using the CTCAE v5.0.

### 2.5 Statistical analysis

Outcomes were compared using paired Student *t*‐test for means, and McNemar and Cochran's *Q* test for proportions. Analyses are conducted using the SAS V9.3 software, with a *p*‐value <0.05 level considered as statistically significant.

### 2.6 Sample size estimation

To provide the ability to calculate a two‐sided 95% exact binomial confidence interval for an 80% efficacy rate with a confidence interval that is plus or minus 10.3% (80% ± 10.3%), that is (69,7%; 90,3%), and taking in account a rate of 17% of patient withdrawal before primary endpoint evaluation, it was considered necessary to include at least 76 patients.

## 3 RESULTS

Between December 2021 and March 2023, 40 patients were included and 37 eligible patients were treated with targeted TMA. At baseline, median (IQR) age was 68 (63–72) years, PSA of 8 ng/mL^6^, ^7^, ^8^, ^9^, ^10^, ^11^ and prostate volume of 45 (34–57) mL on MRI. Twenty‐eight (70%) and 12 (30%) were T1c and T2a stage, respectively. A median (IQR) number of 10^8^, ^9^, ^10^, ^11^, ^12^, ^13^ systematic biopsy cores were performed, with a maximum cancer core length (MCCL) of 6 mm^2^, ^3^, ^4^, ^5^, ^6^, ^7^, ^8^, ^9^. A median number of three^3^, ^4^ targeted cores were performed in the index tumour, with a MCCL of 9 mm^5^, ^6^, ^7^, ^8^, ^9^, ^10^, ^11^, ^12^, ^13^, ^14^. Index tumour characteristics are reported in Table 1.

**TABLE 1:** Prostate index tumour characteristics, at baseline in the 40 patients.

Variable	Median	Interquartile range
Largest axis on mpMRI (mm) (*N* = 40)	11	9.8–13.2
Distance from rectum (*N* = 39)	13	6–20
Distance from far apex (*N* = 39)	16	11–21

Variable	*N*	%
PIRADS (*N* = 39)		
3	0	0
4	36	92
5	3	8
% Gleason grade 4 pattern		
<10%	12	
10–20%	14	
20–30%	3	
30–40%	4	
40–50%	4	
Area	*N* = 40	
Apex	6	15
Median zone mid	23	57
Base	12	30
Prostate region		
PZ‐ant	5	12
PZ‐post lat	15	38
PZ‐post Med	8	19
TZ‐ant	12	31

*Note*: MCCL: Maximum Cancer Core Length; Nb: Number; PZ: peripheral zone; TZ: transitional zone.

### 3.1 Intra and immediate post‐operative outcomes

All patients were treated in an ambulatory setting, under brief general anaesthesia or conscious IV sedation. Intra‐operative outcomes are summarised in Table 2. The transrectal approach was used in 11 patients, in one centre, whereas the four other centres used the transperineal approach. Median pain level, evaluated on analogic visual scale 2 h after the procedure, was of 0/10 (0–1). All patients recovered spontaneous micturition and were discharged the same day.

**TABLE 2:** Intra‐operative outcomes in the 37 patients treated.

Variable	Median	Interquartile range
Nb of focal ablations per patient	3	2–4
Treatment margin applied (mm)	4	3–5
Anaesthesia duration (min)	78	66–90

Variable	*N* (total = *X*)	%
Surgical approach	37	
Transrectal	11	30
Transperineal	26	70
Treatment margins applied (mm)		
0	2	5
1	1	3
3	12	32
4	6	16
5	9	24
6	5	14
7	1	3
10	1	3
Ablation settings applied (*N* = 103)		
12 W – 1 min	0	0
12 W – 2 min	6	6
12 W – 3 min	37	36
12 W – 4 min	39	38
12 W – 5 min	21	20

### 3.2 AEs and functional outcomes

Prostate mpMRI performed at day 7 did not show any peri‐prostatic necrosis or rectal fistula. After a median follow‐up of 6 months,^2^, ^3^, ^4^, ^5^, ^6^, ^7^, ^8^, ^9^, ^10^ 22 (55%) patients experienced 58 AEs. Of them, 32 (for 16 [43%] patients) were considered to be at least possibly related to the procedure (Table 3). Most of AEs (52, 90%) were observed in the immediate post‐operative period. Six (15%) patients experienced an acute urinary retention. Of them, two of were rehospitalised for drainage and monitoring, and another one had a transurethral resection of the prostate (TURP) after 1 month. All severe adverse event (SAE) were resolved at the time of the analysis.

**TABLE 3:** Adverse events.

AEs	*N*	Pts involved	Resolved	Ongoing^a^	Most observed events
Total	58	22	45	13	
SAE	5^b^	3	5	0	AUR
CTCAE 1	43	22	31	12	AUR (4 pts), LUTS (20%), pain (8%), haemospermia (10%), mild haematuria (8%), UTI (8%)
CTCAE 2	13	7	12	1	
CTCAE 3	2	2	2	0	AUR

*Note*: AEs, adverse events; AUR: acute urinary retention; CTCAE, Common Terminology Criteria for Adverse Events.

^a^ At interim analysis.

^b^ Were considered as SAE on the grounds of rehospitalisation for urine drainage (four patients) and TURP (one patient).

**FIGURE 1:**
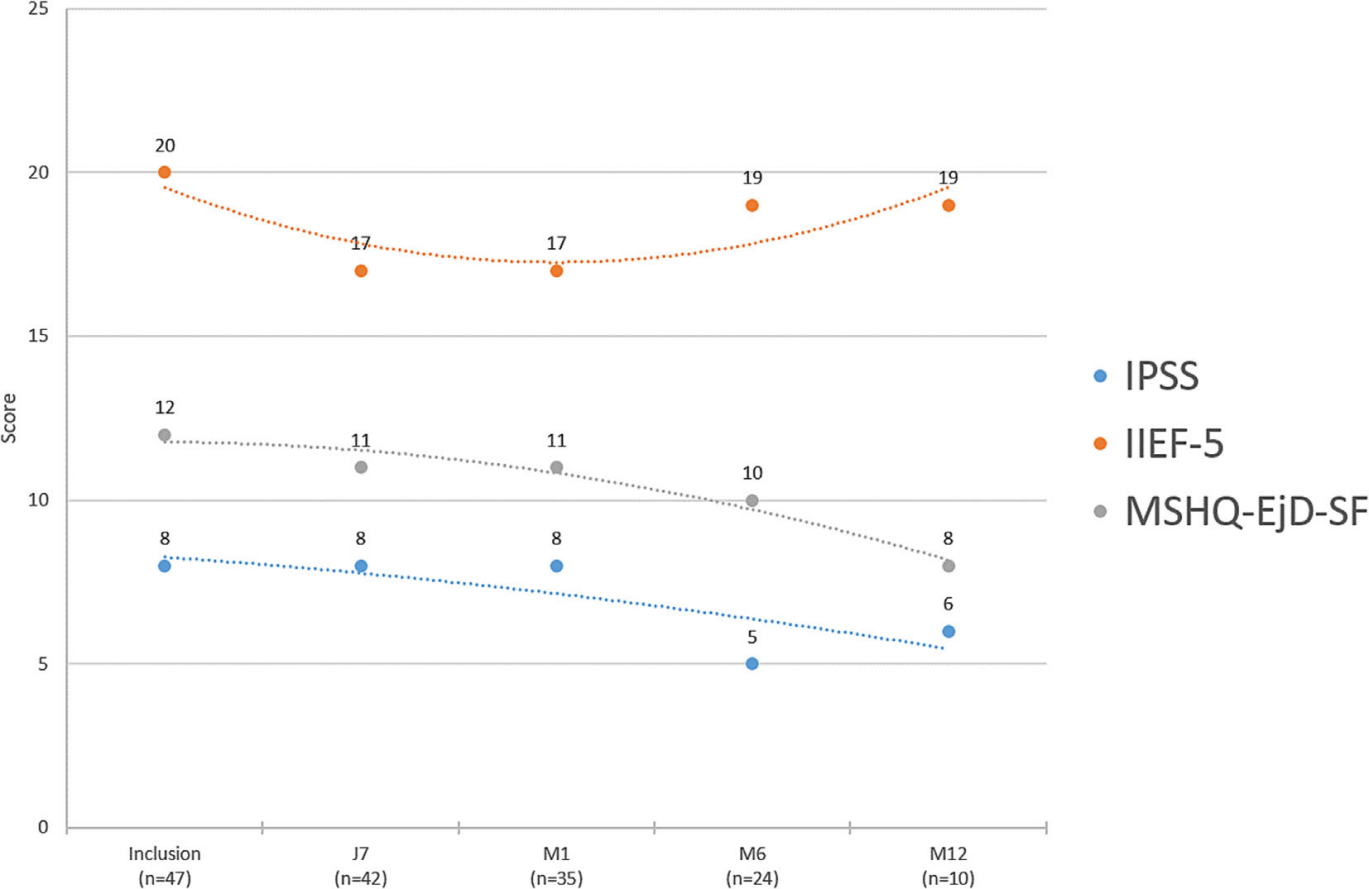
Urinary and sexual functional outcomes after TMA. TMA, targeted microwave ablation.

Figure 1 summarises the evolution of urinary and sexual outcomes. No significant difference is noted at 7 days and at 1, 3 or 6 months post‐operatively as compared to the baseline data.

### 3.3 Oncological outcomes

**FIGURE 2:**
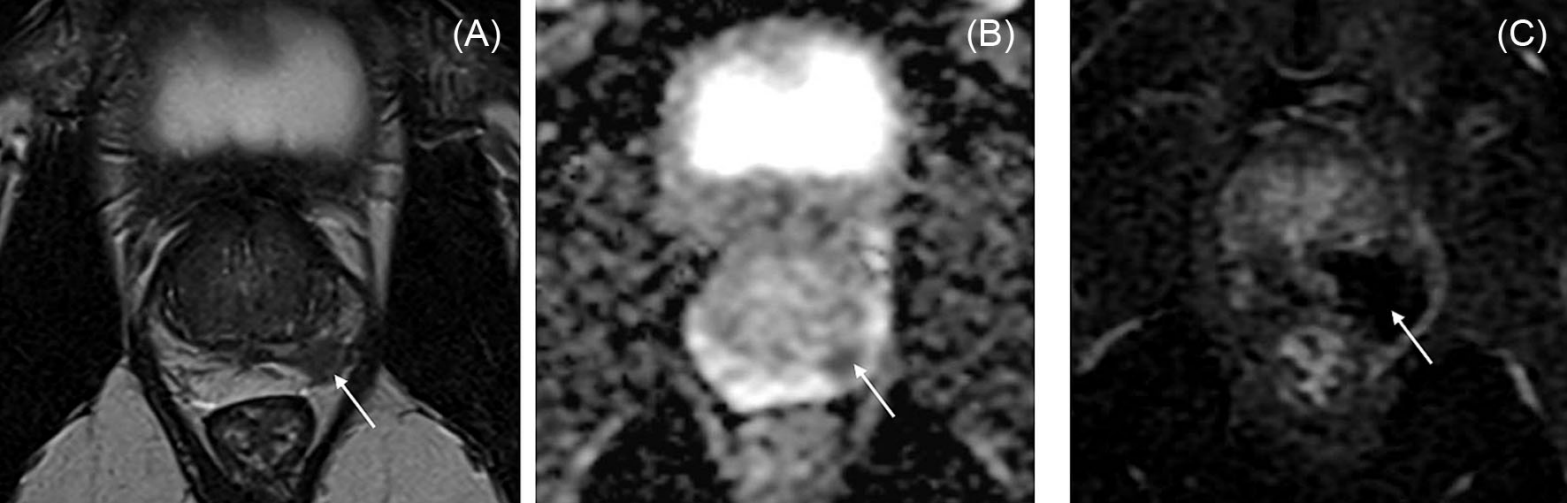
(A) MRI T2 and (B) ADC image showing an index tumour of 12 mm of largest axis (white arrow), treated with two ablations of 4 min (treatment margin set à 4 mm). (C) Dynamic MRI performed 7 days after treatment shows an avascular area (white arrow) covering entirely the index tumour.

Seven days after the procedure, the volume of non‐vascularised prostatic tissue on DCE MRI was available in 37 patients. This non‐vascularised area was visible in 36 patients (97%). It was covering more than 100% of the targeted tumour index, with a median (IQR) value of 6 mL^4^, ^5^, ^6^, ^7^, ^8^ (Figure 2).

**FIGURE 3:**
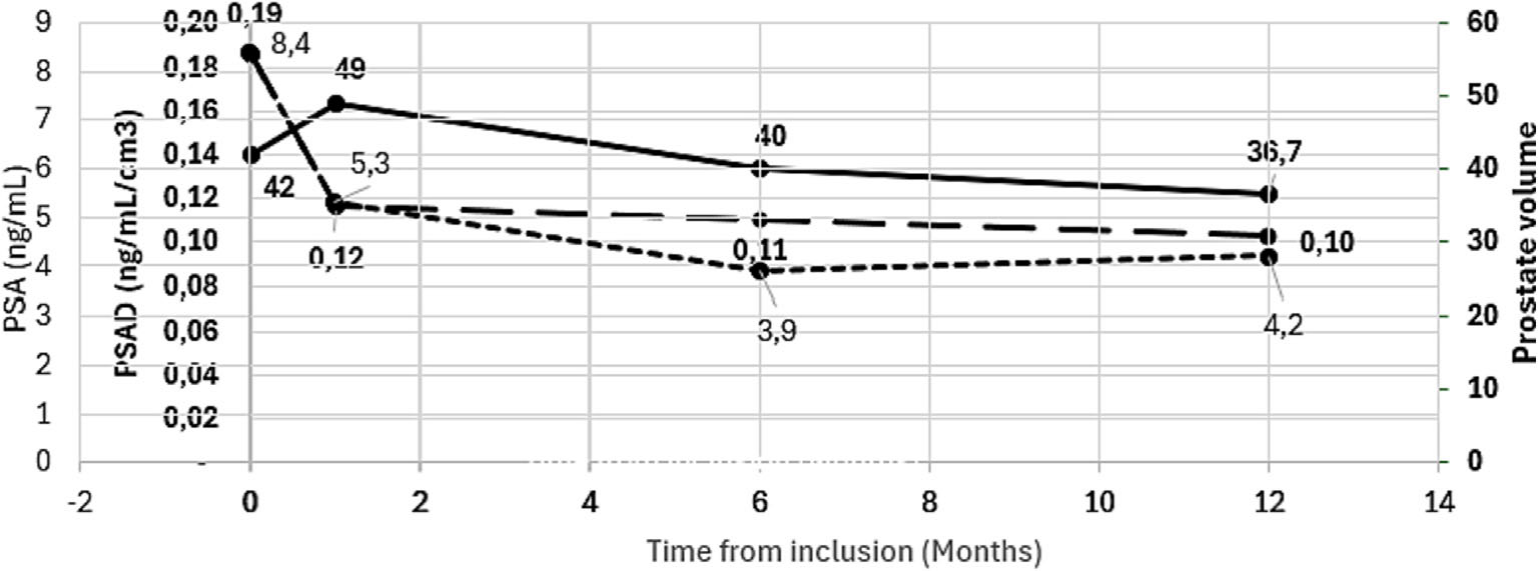
Evolution of total PSA, prostate volume and PSAD. PSA, prostate‐specific antigen.

Evolution of total PSA density, prostate volume, and PSAD is summarised in Figure 3.

## 4 DISCUSSION

This study is the first multicentric phase II evaluation of OBT guided TMA in men with intermediate PCa harbouring a single ISUP 2 index tumour. Three pilot studies were published so far, with encouraging results, suggesting that the ablation zone can be reliably targeted with OBT fusion technology, and then safely ablated with microwaves, using either the transrectal^9^, ^10^ or transperineal^11^, ^12^ approach. In the largest pilot study (15 patients), 21 out of the 23 cancer targets treated (91%) were free of cancer on follow‐up biopsies at 6 months.^11^ None of the pilot studies reported any AE in the next 6 months following the procedure. Moreover, urinary symptoms, uroflowmetry, erectile function, and quality of life scores had no significant difference at 6 months.^9^, ^10^, ^11^, ^12^

The primary endpoint of the present study is to evaluate the oncological efficacy of TMA. However, the aim of this interim analysis was to confirm the precision of ablation on day 7 MRI, as well as the good safety profile of the technique.

Only two investigators had a previous experience in TMA. Nevertheless, because the technique is very similar to OBT‐guided targeted biopsy, the training was rapid for urologists with fusion biopsy experience. OBT‐guidance enables to perform a precise contouring of the index tumour, followed with reliable targeting, and thus to take full advantage of the benefit provided by multiparametric MRI. The use of a dedicated treatment software allows us to visualise the treatment margin that we want to apply to the treatment volume. This margin is set between 1 and 10 mm, according to the investigator's choice. The software also provides a reliable 3D‐view of the treatment zone before and after ablation, which allows us to avoid any cognitive estimation or targeting. Short ablation times (3–5 min per ablation) and small treatment zones (ellipsoid 10‐ to 15‐mm diameter × 20–25 mm long axis) allow for sequential and repeated treatments to cover the tumour area more comprehensively. An interesting aspect of the technique is the possibility to treat any area within the gland, especially through a transperineal approach. A flexible minigrid is used to guide the needle to all regions of the prostate, avoiding injuries to the bulbous urethra (especially for midline tumours) or hitting the pubic bone (which is not uncommon for lateral tumours in large glands).

An extension of the necrosis area beyond the target zone may result in potentially severe AEs, especially if the treatment zone is located close to the rectal wall, to the urethra or to the neurovascular bundles. None of our patients presented such safety issue on prostatic MRI performed 7 days after the procedure. In previous pilot studies, only transient grade 1 complications were observed, including mild haematuria, dysuria or perineal discomfort.^9^, ^10^, ^11^, ^12^ These initial observations were confirmed in most of the cases, with grade 1 or 2 AEs representing 97% of the whole. The most severe AE was urinary retention that occurred in six (15%) patients. One of them had prostatitis which had occurred a few days before. According to a recent review,^6^ urinary tract infection and urinary retention are the far most common grade 2 and 3 complications after high‐intensity focussed ultrasound (HIFU) and cryotherapy. In a recent cohort of 55 men treated with partial gland cryoablation, urinary retention needing a catheterisation was reported in nine (16%) patients.^14^ In their series of 153 patients treated with focal HIFU, Bass et al.^15^ reported Clavien–Dindo grade 1 in 35 men (23%), grade 2 in 12 men (8%) and grade 3 in four men (2.6%). Urinary retention was also reported as the most common AE (20 men, 13.1%). In another recent series of focal HIFU, Dellabella et al.^16^ even reported that 8% of the patients required transurethral resection of the prostate for recurrent urinary retention. In our study, none of the patients who presented a urinary retention were treated transrectally. This may suggest that the transperineal approach is more likely to provoke urinary retention. However, a recent meta‐analysis comparing transrectal to transperineal biopsies showed no significant difference in acute urinary retention between the two approaches.^17^ There are probably various factors leading to such complication, including the degree of previous benign prostatic obstruction, the proximity of the urethra from the ablative zone and intercurrent urinary tract infection.

The primary outcome of this prospective and ongoing phase II study is the presence of ISUP 2 cancer in the treated zone, evaluated with targeted biopsy at 12 months. Post‐FT surveillance consensus recommendations suggest that small volume 3 + 3 or very small volume (< 0.2‐cc or < 7‐mm diameter) 3 + 4 are acceptable in the treated zone at longitudinal follow‐up and may not need additional treatment.^18^, ^19^ At this point of our evaluation, only PSA data are available and used as a surrogate for tumour progression evaluation. Total PSA is relevant for patient follow up during active surveillance but may be much less reliable after any focal treatment.^6^, ^18^ In the latter, PSA kinetics may not reflect directly the efficacy of treatment and also the reduction in prostate size following tissue ablation. PSA nadir has been found to have a poor correlation with residual cancer on biopsy.^20^ In our evaluation, a new estimation of prostate volume at 6 months is possible using MRI. We observe a reduction in prostate volume that may have affected total PSA level. The estimation of PSA density enabled to correct this possible bias for each patient. The reduction in PSA density we observe at 6 months may reflect more reliably the oncological efficacy of TMA. MRI may also have a value for assessing the efficacy of FT, but its role remains to be defined. At this time of analysis, MRI images were compared between day 7 and baseline in the 25 first patients. The targeted tumour index had completely disappeared and was covered with non‐vascularised tissue on dynamic contrast‐enhanced (DCE) MRI. Shrinkage, disappearance or no enhancement of the initial target may be associated with absence of clinically significant cancer on control biopsies.^6^, ^21^ In our study, all MRI images will be analysed and compared between baseline, 7 days, 6 and 12 months to better understand the expected changes associated with successful treatment.

Our study is limited by the absence of comparator. This drawback is, however, common in phase II FT evaluation and because of the morbidity of standard treatments. The only direct comparison was performed with photodynamic therapy (Tookad) versus active surveillance.^22^ Our work is, however, the first multicentric evaluation of this innovative OBT‐guided focal treatment. Although limited by the small number of patients, recruitment is ongoing and should reach 76 patients in the next few months. The evaluation of the primary outcome will be crucial to confirm the relevance of the technique.

## 5 CONCLUSIONS

The preliminary results of VIOLETTE study suggested that OBT‐guided TMA is precise and safe in patients with intermediate risk localised prostate cancer. Further inclusions and follow‐up are needed to assess oncological outcomes.

## AUTHOR CONTRIBUTIONS

All authors contributed to the study as investigators and participated in data collection, analysis and manuscript writing and reviewing.

## CONFLICT OF INTEREST STATEMENT

Nicolas Barry Delongchamps and Julien Anract: proctoring activity for Koelis.

The other authors have no potential conflicts of interest to declare.
